# Role of imaging and endovascular radiology in endoscopically missed Dieulafoy’s lesion of stomach – A case report with review

**DOI:** 10.1259/bjrcr.20210117

**Published:** 2022-03-09

**Authors:** Divyesh Dadhania, Jineesh Valakkada, Anoop Ayyappan, Santhosh Kannath

**Affiliations:** 1 Department of Imaging Sciences and Interventional Radiology, Sree Chitra Institute of Medical Sciences, Trivandrum, Kerala, India

## Abstract

Dieulafoy’s lesion is an uncommon cause of life-threatening gastrointestinal bleed from a dilated and tortuous submucosal artery. With the advent of endoscopy-guided intervention, the mortality of the condition has reduced significantly from 80 to 8%. Imaging plays a vital role in diagnosing them in endoscopically negative cases. Endovascular management can also be offered for unidentified lesions or failed endoscopic treatment. We report a middle-aged male with acute hematemesis where endoscopy was unable to reveal the source of the bleed. Contrast CT detected the lesion, which was embolised by endovascular route. The clinical details, imaging appearance and treatment of this uncommon lesion is presented.

## Introduction

Dieulafoy’s lesion (DL) is an uncommon life-threatening cause of gastrointestinal (GI) bleed. It consists of a tortuous, aberrant submucosal artery in the gastrointestinal tract, which penetrates, erodes and eventually perforates the mucosa over time, causing severe gastrointestinal bleeding. Although endoscopy is the first-line option for the diagnosis and management of such lesions, it can fail to detect the lesion if the bleeding is intermittent and in overlooked areas like the fundus of stomach. Cross-sectional imaging can help in the diagnosis of such lesions as well as planning for endovascular intervention. We report a middle-aged male with acute hematemesis due to DL embolised by the endovascular route wherein contrast CT detected the lesion which was missed by endoscopy.

## Case

**Figure 1.**
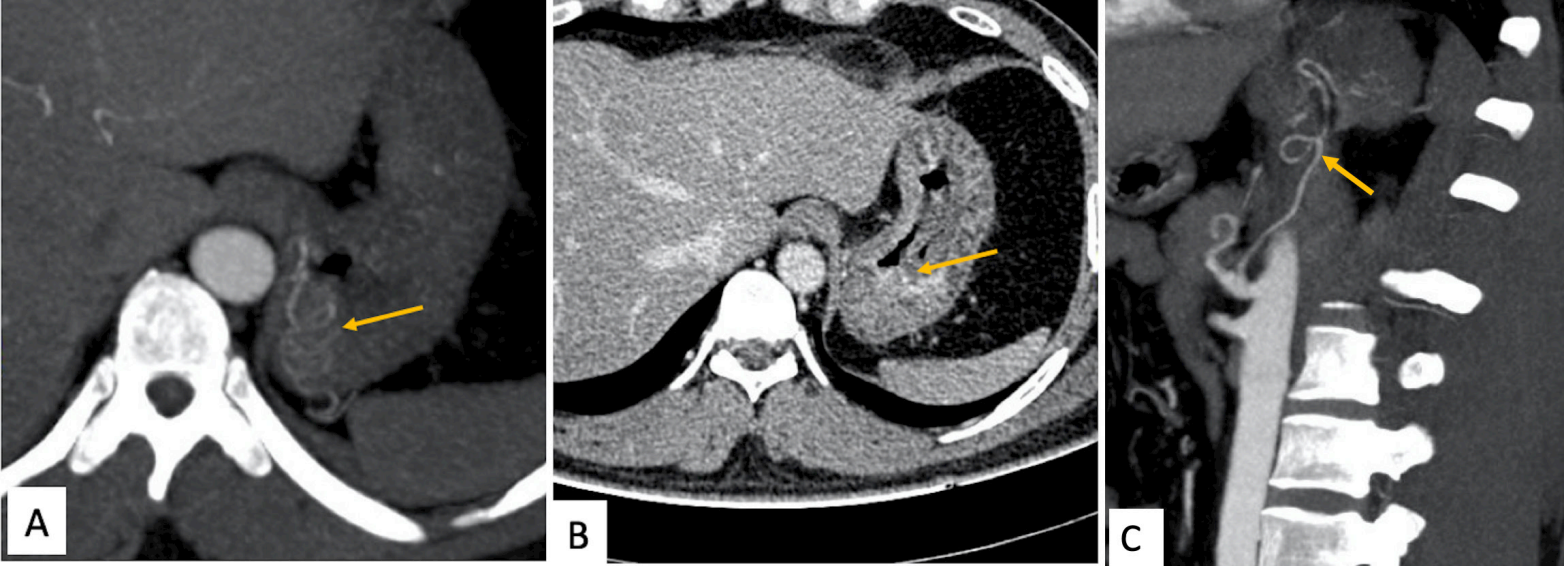
Arterial phase of contrast CT (**A and C**) showing abnormal tortuous vessels (arrow)in the gastric fundus, which shows an active leak in the venous phase (arrows in B).

**Figure 2.**
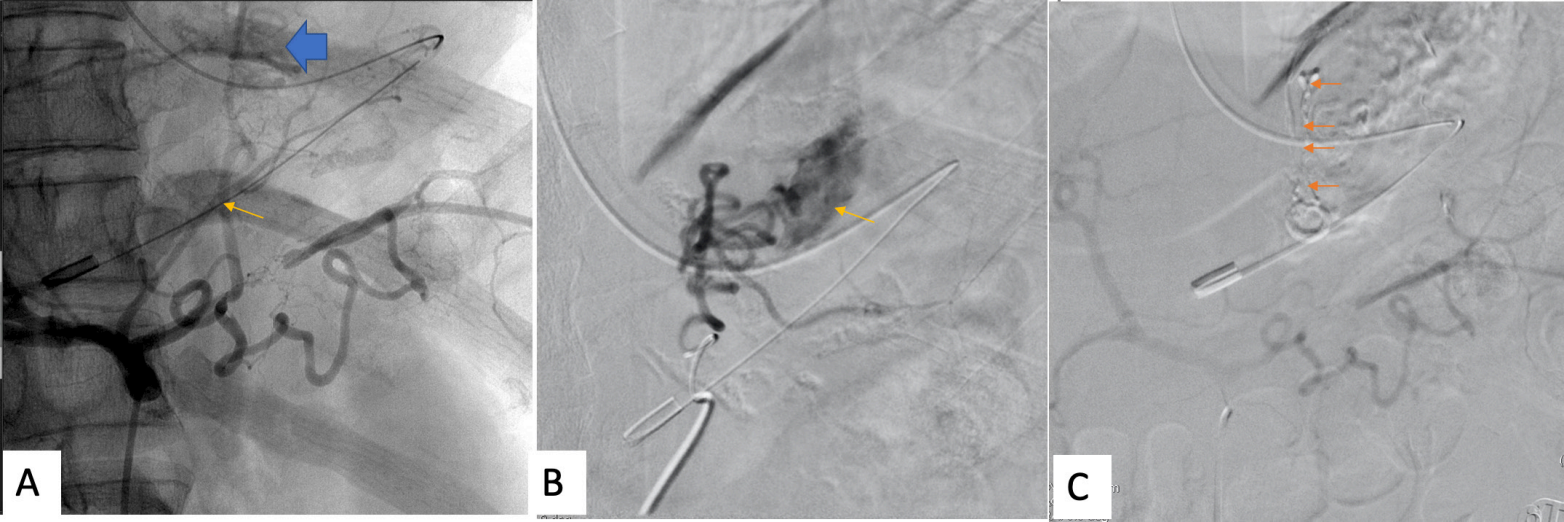
Coeliac angiogram (**A**) showing a tortuous vessel from the left gastric artery (thin arrows) supplying the lesion (thick arrows in A). Super selective distal angiogram of the left gastric artery(B) showed the lesion as a tortuous artery with active leak from the gastric fundus (arrow) with no early draining vein. The lesion was embolised using 30% N-Butyl cyanoacrylate (arrows in C) with a post-embolisation angiogram showing complete obliteration of the lesion.

A 35-year-old male without comorbidities presented to the emergency department with acute hematemesis and melena for two days duration. He denied alcohol abuse, smoking, peptic ulcer disease or ingestion of any NSAID. Clinical examination revealed pallor with a heart rate of 102/min and blood pressure 98/60 mm Hg. Haemoglobin was 7.2 gm% with normal INR (1.3) and platelets (130 × 10^9^/L). After stabilising with i.v. fluids and packed red blood cells, emergency endoscopy was done which showed fresh blood in the stomach. However, the source of bleed could not be localised. A repeat bout of fresh blood prompted an emergency CT angiogram which showed a tortuous tangle of dilated blood vessels in the fundus of stomach arising from the left gastric artery with active extravasation of contrast (Figure 1). Given the location and configuration of the vessel, a diagnosis of Dieulafoy’s lesion was considered and planned for endovascular management. Angiogram of the coeliac artery corroborated with CT and showed a tortuous vessel from the left gastric artery without an early draining vein (Figure 2). In view of marked tortuosity of the vessel and distal location of the abnormality, a decision to embolise the feeding artery with 30% n–Butyl cyanoacrylate(glue) was made. Through a 1.7F microcatheter, the proximal curves of the vessel were negotiated, and embolisation was performed ensuring no collateral flow into the lesion (Figure 2C). The patient responded well with vitals getting stabilised within 30 min and resolution of melena in 2 days. Six months clinical follow-up showed no rebleed.

## Discussion

Dieulafoy’s lesion variably known as persistent calibre artery, gastric arteriosclerosis or cirsoid aneurysm represent 1–2% of the causes of upper gastrointestinal with Incidence increasing by the extensive use of endoscopy.^1^ It is commonly seen in stomach along the lesser curvature (70–80%) within 6 cm of gastro-oesophagal junction followed by duodenum (15%), colon (5%) and rarely in oesophagus and bronchus.^2^ It affects all age group with male-to-female ratio of 2:1. The mean age at presentation is within the fifth decade of life (Range 50–70 years). Associations with diabetes mellitus, hypertension, chronic ischaemic heart disease, NSAID use, neurological disorders, liver diseases, and respiratory and renal failure have been described.^3^

They are persistent/large calibre submucosal arteriole arising from visceral vessels. Although histologically normal, these vessels are highly tortuous (Figure 2B) and lack distal tapering (measuring 2–3 mm throughout) passing indolently in submucosa. The occurrence near GEJ may be due to the peculiar blood supply to the lesser curve of the stomach as these vessels arise directly from the arterial chain in the lesser curve while the arterial supply to the remainder of the stomach is derived from a submucosal plexus of larger vessels. Although over a hundred years have passed since the first description of this lesion by the French surgeon Dr George Dieulafoy, the exact mechanisms causing the tortuosity and the persistence of the large-sized submucosal arteries remain unknown.^4^ The theories of the cause of rupture include pulsations of the abnormally large artery disrupting the mucosa and exposure of the artery to gastric/bowel contents, gastric “wear and tear” promoting the formation of an arterial thrombus that causes necrosis and age-related atrophy. The bleeding can be severe and intermittent, resulting in difficulty in diagnosis.^5^ The site of bleed is usually devoid of any inflammation or mucosal abnormality camouflaging endoscopic visualisation during an intermittent bleed.

Patients are typically asymptomatic before presenting with acute, profuse GI bleeding, which can manifest as hematemesis, melena, or haematochezia. Since the mortality rate due to bleed reduces drastically from the untreated lesion (80%) to treated ones (8%), accurate diagnosis and management is vital.^5,6^ Endoscopy can reveal lesion in up to 70% of cases at the time of bleed, with repeat endoscopy increasing the sensitivity to about 90%.^7^ They can be mistaken for arteriovenous malformations, gastric antral vascular ectasias, angiodysplasias or Mallory-Weiss tear on endoscopy. Endoscopy can be challenging in active bleeding (obscuring the bleed site), in intermittent bleeds and if the lesion occurs at ‘blind spot’ like the fundus of stomach. CT angiogram (without oral contrast) can bridge this diagnostic gap by visualising the tortuous vessels.^1^ Although there is sparse literature regarding the role of CT angiogram in Dieulafoy’s lesion, the advantage of CT is that it can show the abnormal, persistent calibre vessel in arterial phase even with lack of active extravasation/bleed.

Although no standard guidelines are available, the lesion is managed primarily by endoscopy with reported success rate in excess of 80%.^8^ Endoscopic haemostatic procedures can be (a) thermal using heat probe or argon plasma coagulation; (b) regional injection of epinephrine or sclerotherapy; and (c) mechanical – banding and haemoclip with combined therapy is more effective than monotherapy.^9^ Since the risk of re-bleeding from endoscopically treated Dieulafoy’s lesion has been reported to range between 9 and 40%, there is a need to closely follow patients in the post-procedural period. Endovascular options can be attempted in failed endoscopy or lesions beyond the reach of endoscope. Digital subtraction angiogram reveals a tortuous vessel at the culprit site with or without contrast leak. The absence of an early draining vein in angiogram is a vital sign to differentiate the lesion from arteriovenous malformation and angiodysplasia, which warrants different treatment. Many embolic agents have been tried, including gel foam, glue, coils and PVA particles^7,10^ with a success rate of 60–70%. Although endoscopic glue injection has been reported in the literature for DL with success rate of 80–90%, endovascular glue has been sparingly used probably due to fear of gastric ischaemia.^11,12^ However, since lesion usually does not supply the mucosa, the use of glue is safe in such lesions without risk of ischaemia. Glue has the advantage of percolating distally in the tortuous vessels challenging to navigate, resulting in complete occlusion of the bleeder resulting in prompt significant response and reducing recurrence rate. Since the lesion is highly tortuous, smaller size microcatheters (<2 F) are required to navigate the distal site.^13^ Proximal embolisation with coil results in filling via collateral, resulting in high recurrence rate of 60–70% requiring repeat endoscopy or surgery (since coil prevents renavigation in the index lesion).^7^ Surgical resection is currently reserved for the 5% of cases that are refractive to endoscopic or angiographic methods. The long-term prognosis of a properly treated Dieulafoy’s disease is good with a recurrence of 10–15% and a mortality of 8–10%, which is identical between the three modalities^14^

## Learning objectives

- Dieulafoy’s lesion (DL) needs to be considered in patients presenting with severe upper GI bleed without evidence of portal hypertension or peptic ulcers.
- CT angiography has the advantage over endoscopy in DL in detecting abnormal vessels even in the absence of active bleed.
- Angioembolisation can be attempted in cases of failure of therapeutic endoscopy.
- N-butyl cyanoacrylate is a safe agent for these lesions to attain complete angioembolisation and to prevent recurrence.
